# Expression of Vimentin in hair follicle growth cycle of inner Mongolian Cashmere goats

**DOI:** 10.1186/s12864-017-4418-7

**Published:** 2018-01-10

**Authors:** Nai Rile, Zhihong Liu, Lixia Gao, Jingkai Qi, Meng Zhao, Yuchun Xie, Rui Su, Yanjun Zhang, Ruijun Wang, Jie Li, Hongmei Xiao, Jinquan Li

**Affiliations:** 1 College of Animal Science, Inner Mongolia Agricultural University, Hohhot, 010018 China; 2 Key Laboratory of Animal Genetics, Breeding and Reproduction, Inner Mongolia Autonomous Region, Hohhot, China; 3 Key Laboratory of Mutton Sheep Genetics and Breeding, Ministry of Agriculture, Hohhot, China; 4 Engineering Research Center for Goat Genetics and Breeding, Inner Mongolia Autonomous Region, Hohhot, China; 5 School of Life Science, Inner Mongolia University for The Nationalities, Tongliao, 028000 China; 6 Inner Mongolia Academy of Agriculture and Animal Husbandry Sciences, Hohhot, 010018 China; 7 Baotou Light Industry Vocational Technical College, Baotou, 014000 China

**Keywords:** Cashmere goat, Skin, Hair follicle, Growth cycle, Vimentin

## Abstract

**Background:**

The growth of Inner Mongolian Cashmere goat skin hair follicle exhibits a periodic growth pattern. The hair growth cycle is distinguished as telogen, anagen, and catagen stages. The role of vimentin in the growth process of hair follicles is evident. To elucidate the mechanism underlying the vimentin activity in the growth cycle of hair follicles, transcriptome sequencing and liquid chromatography-tandem mass spectrometry were used to obtain the nucleic acid and amino acid sequences of *VIIM* gene and vimentin. The amino acid and nucleic acid sequences were analyzed by comparison. Real-time quantitative PCR, Western blot, and immunohistochemistry analyzed the expression level and sites of vimentin in the three growth stages of the Inner Mongolia Cashmere goat skin samples.

**Results:**

*VIM* gene cDNA, obtained by transcriptome sequencing, was aligned against that of the *Capra hircus VIM* gene. The amino acid sequence of vimentin revealed a high similarity rate across other species. The expressions of both *VIM* gene and vimentin were highest during the growth period and lowest in the rest period. Furthermore, vimentin was primarily expressed in the outer root sheath of the hair follicle as assessed by staining.

**Conclusions:**

The sequences of the gene and protein are similar to that of other species and identical to *Capra hircus*. However, the expression of *VIM* and vimentin was proportional to that of the growth of hair follicles. And vimentin expressed only in the outer root sheath of hair follicles. Thus, vimentin was speculated to participate in the regulation of the hair follicle growth cycle by affecting the outer root sheath.

## Background

The fleece of Inner Mongolia Cashmere goat can be classified as heterogeneous. Cashmere is a product of the secondary hair follicle and wool is a product of the primary hair follicle. And they undergoes a periodicity comprising of three stages anagen, catagen, and telogen [1]. The periodic changes in the hair follicle are attributed to the complex and orderly biochemical processes including the interaction and inhibition of multi-molecule particles in the skin. Thus, the expression level of proteins involved in these processes varies correspondingly.

Vimentin is a type III intermediate filament (IF) protein, which is expressed in the whole cytoplasm, except the organelles and nucleus [2, 3]. It is encoded by the *VIM* gene located on chromosome 13 [4]. The length of the DNA sequence of the *VIM* gene is approximately 10 kb. The full length of the cDNA is about 1848 bp, and the open reading frame is about 1401 bp [5], containing 9 exons that encode 466 amino acids. The protein sequence of vimentin is highly conserved across evolution [5]. The main function of this protein is the maintenance of cell morphology [6–8], signal transmission between cells [9–11], regulation of cell differentiation and proliferation [12, 13], and participation in cell stress response [14]. Vimentin also plays a major role in the development of tumors [15, 16] and viral infections caused by Herpes virus [17], immunodeficiency virus [18], and other viruses. In addition, as an IF protein, vimentin might also be related to the transport of mRNA [19]. A previous study showed that the expression level of vimentin decreased gradually with the growth of a fetal rat skin [20]. Another study proposed that the highest expression of vimentin was observed in the anagen stage of the growth cycle of the mouse skin follicle [21]. Furthermore, a significant difference in vimentin expression levels was observed during the stages of the development cycle of the skin follicle in Inner Mongolia Cashmere goat; the expression was high during the growth period [22]. In summary, vimentin plays a vital role in the growth process of the development cycle of the skin follicle.

## Methods

### Animals

The Experimental Cashmere goats were obtained from Aerbasi White Cashmere Goat Breeding Farm in Inner Mongolia, China. These goats belong to a breed of inner Mongolian Cashmere goats [23]. Three adult individuals (females, 2-year-old) were randomly selected for biological replicates. Skin samples were collected from the back of the goat during the hair follicle developmental stages of telogen (March), anagen (July), and catagen (December). The samples were transported to the laboratory in liquid nitrogen and preserved at −80 °C.

### Determination of mRNA sequence of *VIM* gene

The total RNA from the skin tissue was extracted using TRIzol (Takara, Dalian, China). Then, the samples were ground in liquid nitrogen, according to the manufacturer’s instructions. The concentration and purity of RNA were detected using a Beckman DU 800 Nucleic Acid-Protein Analyzer (Beckman-Coulter, Fullerton, CA, USA). The integrity of the total RNA was analyzed by 1% agarose gel electrophoresis. Furthermore, the extracted RNA was synthesized into cDNA (Illumina TruSeq™ RNA sample preparation kit, USA) for transcriptome sequencing. The next-generation sequencing of the cDNA was performed using an Illumina HiSeq2000 platform; the sequencing length was 2 × 100 bp.

### Real-time quantitative PCR

The cDNA obtained above was used for real-time quantitative PCR (RT-PCR) analysis. Gene-specific primers were designed using the software Primer 3.0 and synthesized by Sangon Biotech Co., Ltd. (Shanghai, China). The primer sequences and fragment size are listed in Table 1. The quantitative reverse transcription RT-PCR was performed using the PrimeScript RT Reagent Kit (TaKaRa) in a 20-μL reaction volume with 10 μL of 2× SYBR Premix Ex Taq II (TaKaRa), 0.4 μL ROX, and 0.5 μL each primer, and 7.2 μL DNase/RNase-free water. The reaction was assessed on a Bio-Rad IQ5 Multicolor RT-PCR Detection System (Hercules, CA, USA). We selected the β-actin as the internal contral. The conditions for quantitative RT-PCR are listed in Table 2. The RT-PCR analysis was performed using the 2^−ΔΔCT^ method. Statistical analysis was conducted using the GLM method of the SA software (version 9.0; SAS Institute, Cary, NC, USA). The values are represented as the mean ± standard deviation. A significance level of 0.05 was used.

**Table 1:** Primer sequences and fragment size of Cashmere goat *VIM* gene and β-actin

Gene Name	GenBank number	Sequence of primer	Products size
*VIM*	XM_005688054.1	F: TTTCTCCACGCCTCCAGTT R: ATGTCTTCGGCCAGGTTGT	296 bp
*β-actin*	NM_001009784.1	F: GGCAGGTCATCACCATCGG R: CGTGTTGGCGTAGAGGTCTTT	158 bp

**Table 2:** Parameters for quantitative RT-PCR

Temperature	Time	Cycles
94 °C	510 min	
94 °C	30 s	40
62 °C	30 s	
72 °C	30 s	

### Acquisition of vimentin sequence and multiple sequence alignment

An equivalent amount of Inner Mongolia Cashmere goat skin samples were ground in liquid nitrogen, followed by the addition of 1% sodium dodecyl sulfate(SDS)to the lysate. The mixture was incubated at room temperature for 20 min, followed by ultrasonication for 2 min and centrifugation at 12,000 rpm for 15 min at 4 °C. The total protein concentration in the supernatant was determined using the BCA (Bioteke,Bei Jing, China) kit. The protein was hydrolyzed by trypsin, and peptides were analyzed by Triple TOF 5600 (AB Sciex, USA). The protein candidates were searched by software Protein Pilot 4.0. The sequences of vimentin in *Capra hircus*, *Ovis aries*, *Bos taurus*, *Homo sapiens*, and *Mus musculus* were obtained from NCBI/Protein and compared to that of NCBI/BLAST. The three-dimensional structure of vimentin was constructed by the comparative protein modeling program SWISS-MODEL.

### Western blot

0.08 g of skin samples were homogenized in liquid nitrogen, followed by the addition of cell lysis buffer and PMSF (Beyotime, China) to extract total protein. Subsequently, the tissue samples were placed in an ice bath for ultrasonication, incubated for 30 min, and centrifuged as described above. The total protein was estimated in the resulting supernatant using the BCA kit (Bioteke). An equivalent of 30 μg protein was separated by 12% SDS-polyacrylamide gel electrophoresis (SDS-PAGE), followed by semi-dry transfer to the PVDF membrane (PALL, New York, USA). The PVDF membrane was blocked using 5% skimmed milk at room temperature for 2 h, followed by probing the membrane with mouse monoclonal anti-vimentin antibody (Abcam, Cambs, UK. 1:500) and rabbit polyclonal anti-β-actin antibody (Abcam. 1:1,000) overnight at 4 °C. Subsequently, the membrane was washed with phosphate-buffered saline with tween20 (PBST) and incubated with fluorescent-labeled goat anti-mouse secondary antibody and goat anti-rabbit secondary antibody (LI-COR Biosciences Inc., Lincoln, NE, USA. 1: 3,000) at 37 °C for 1 h. Finally, the membranes were washed, and the immunoreactive bands examined using LI-COR® Odyssey near-infrared imager (LI-COR Biosciences Inc).

### Immunohistochemistry

Fresh skin samples of Inner Mongolia Cashmere goat were obtained during catagen, telogen, and anagen and fixed in 4% paraformaldehyde for 24 h. Following dehydration in pure alcohol, the sections were placed in benzene to replace ethanol prior to paraffin-embedding. The slices of tissue samples (8-μm-thick) were incubated with xylene for dewaxing, subjected to gradient alcohol hydration, and incubated at room temperature in 3% hydrogen peroxide (H_2_O_2_) to inactivate the endogenous catalase. Next, the specimens were flushed with phosphate-buffered saline (PBS), antigen retrieval was carried out in citrate buffer, and blocking with 5% BSA at room temperature for 1 h. Subsequently, the samples were incubated overnight at 4 °C with mouse monoclonal anti-vimentin antibody (Abcam. 1:250); 5% albumin from bovine serum (BSA) was used as a negative control. Then, the samples were washed with PBS and incubated with the HRP-labeled goat anti-mouse secondary antibody ((Beyotime. 1:500) for 1 h at 37 °C. Finally, the sections were stained with hematoxylin and observed under a microscope.

## Results

### Analysis and determination of the mRNA sequence of *VIM* gene

**Fig. 1:**
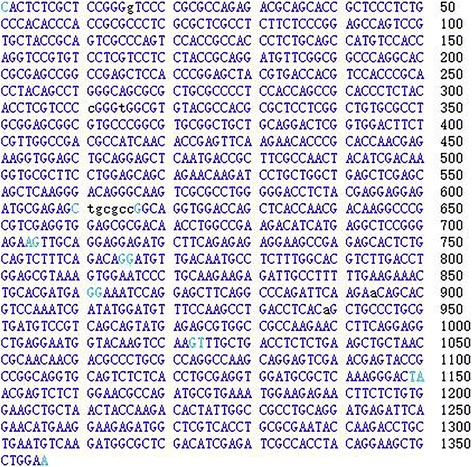
Transcriptome sequencing result of *VIM* gene. The transcriptome sequence of *VIM* gene analysis using Genome Browser shows that the sequence consists of six exons. The pale blue denotes the boundary of each exon

**Fig. 2:**
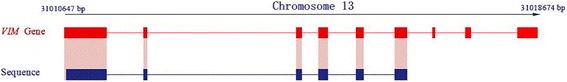
Comparison between the transcriptome sequencing result of the *VIM* gene and *Capra hircus VIM* gene based on the NCBI/GENE. The transcriptome sequence was compared with the *Capra hircus VIM* gene from the GENE database of NCBI. The boxes in the figure represent the exons, and the lines between them represent the introns. The red color indicates the *VIM* gene in the NCBI, while the blue color indicates the sequence identical to that of the transcriptome sequence of the *VIM* gene

The transcriptome sequencing result showed that the length of *VIM* gene sequence was 1356 bp. The results of the sequence analysis using the Genome Browser are illustrated in Fig. 1. The transcript of the *VIM* gene consists of six exons. The blue color of the figure represents the exons, whereas the pale blue denotes the exon boundary. As shown in Fig. 2, the matching rate between the sequence and the *Capra hircus VIM* gene from NCBI/GENE was 100%. The *Capra hircus VIM* gene begins to coincide with the transcript at the position of 48 bp. The squares in Fig. 2 represent the exons, and the lines between them represent the introns. The red color indicates the *Capra hircus VIM* gene from the NCBI; the blue color represents the sequence position at which the transcriptome sequence is identical to that of the *Capra hircus VIM* gene.

### Differential expression of the *VIM* gene in the skin follicle growth cycle of inner Mongolian Cashmere goats

**Fig. 3:**
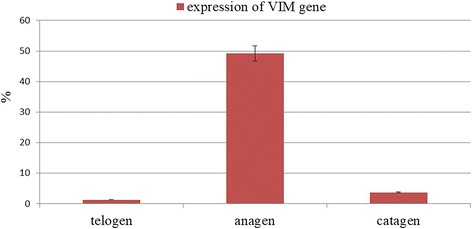
Relative expression levels of the *VIM* gene in the stages of the skin follicle cycle. The RT-PCR was performed using the 2^−ΔΔCT^ method. The values are shown as the mean ± standard deviation. A significance level of 0.05 was used

In this study, RT-PCR analyzed the differential expression of *VIM* gene in the skins during the stages of telogen, anagen, and catagen of Inner Mongolia Cashmere goat (Fig. 3). The difference was significant (*P* < 0.01), and the expression level of *VIM* gene was the highest in the growth period and lowest in the resting period.

### Acquisition of vimentin amino acid sequence and multiple sequence alignment

**Fig. 4:**
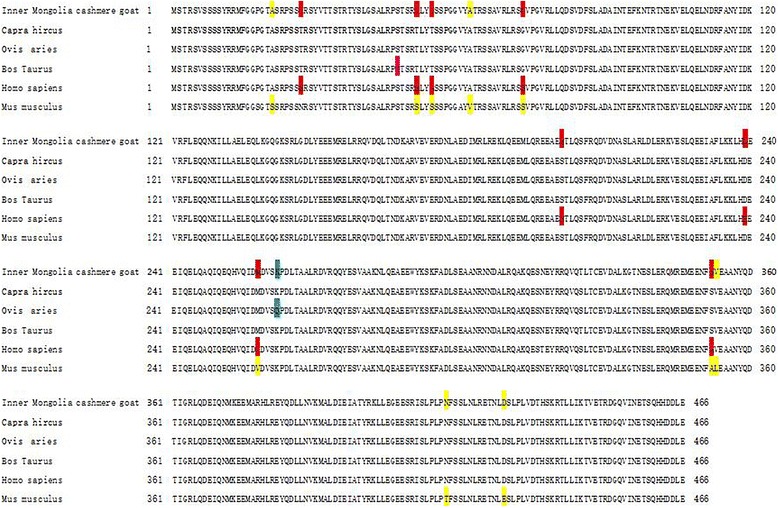
Multiple sequence alignment of vimentin. The amino acid sequences of vimentin were analyzed by multiple sequence alignment. The blue mark represents the differentially expressed sequence of vimentin between Inner Mongolia Cashmere goat and *Ovis aries*. The red mark indicates the site with a difference from that of *Homo sapiens*, and the yellow mark represents the site with a difference in the sequence from that of *Mus musculus*

**Fig. 5:**
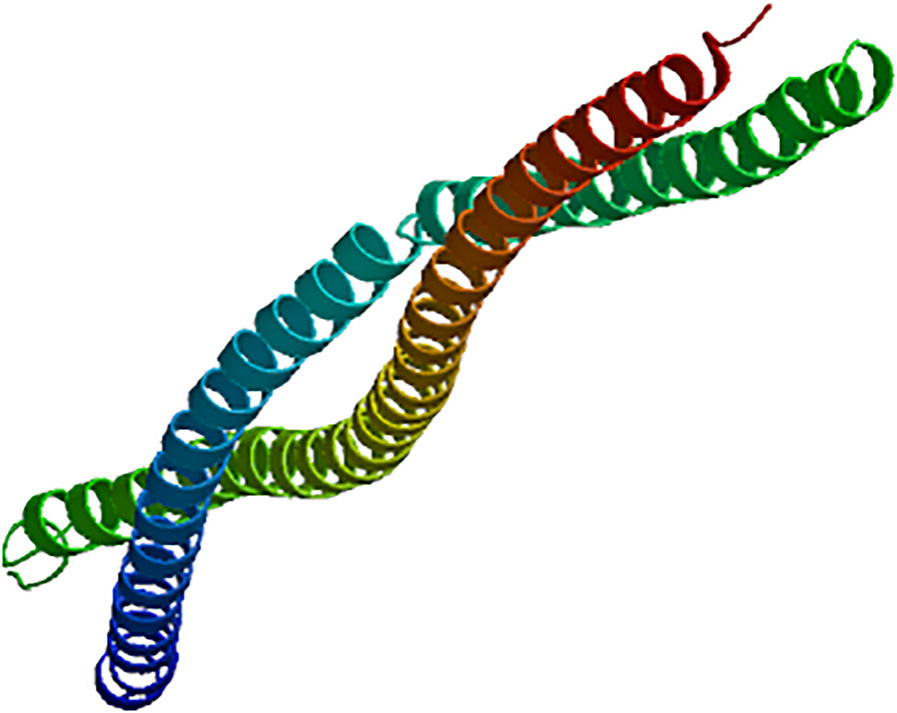
Three-dimensional structure of vimentin. The three-dimensional structure of vimentin predicted based on the amino acid sequence

The length of the amino acid sequence of skin vimentin of Inner Mongolia Cashmere goat was 466 amino acids. Importantly, the length of the vimentin sequence was identical to that with *Capra hircus*, *Ovis aries*, *Bos taurus*, *Homo sapiens*, and *Mus musculus* obtained from NCBI/PROTEIN. The sequence alignment using NCBI/BLAST revealed that the amino acid sequence of vimentin from Inner Mongolia Cashmere goat was 100% identical to that of the vimentin from *Capra hircus,* 99%, 99%, 98%, and 97% identical to that from *Bos taurus*, *Ovis aries*, *Homo sapiens*, and *Mus musculus* (Fig. 4), respectively. The blue mark in Fig. 4 represents the differentially expressed sequence of vimentin between Inner Mongolia Cashmere goat and *Ovis aries*. The red mark denotes the site difference in expression from that of *Homo sapiens*, and the yellow mark represents the site with a difference from the sequence of *Mus musculus*. The three-dimensional structure of vimentin (144–249 amino acids) was established by the comparative protein modeling program SWISS-MODEL (Fig. 5).

### Western blot analysis

**Fig. 6:**
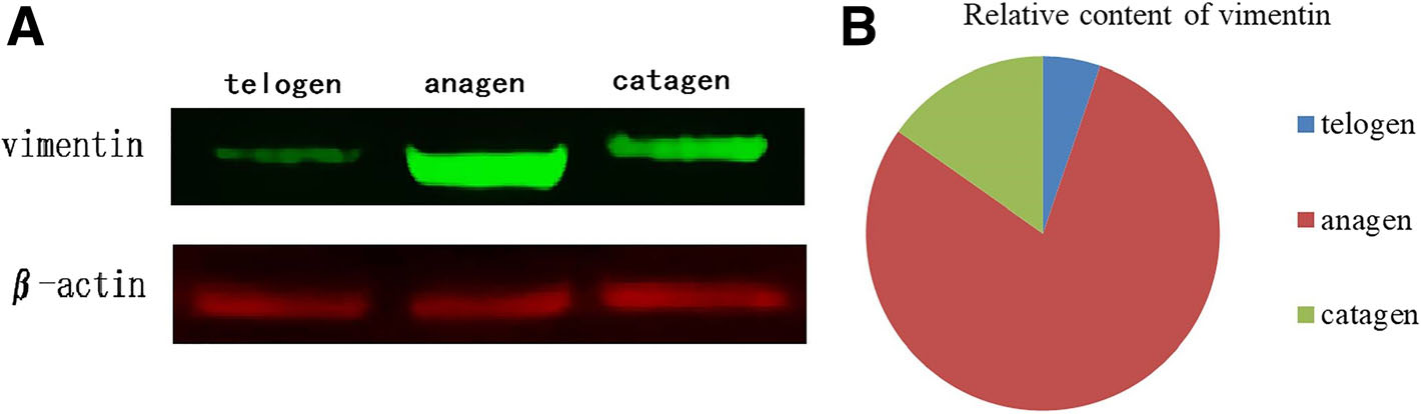
Protein expression by Western blot. **a** Results of Western blot analysis using specific antibodies on protein extracts prepared from Inner Mongolia Cashmere goat skin samples; **b** The relative content of the target protein was the gray-level ratio of the target protein to the internal controls

The expression level of vimentin in the skin of Inner Mongolian Cashmere goat was detected by Western blot. The gray-level ratio of the target protein and the internal controls was used as the index for estimating the relative content of the target protein. Fig. 6a demonstrates the vimentin expression in the skin during all the three periods, and the difference in the expression was extremely significant. Figure 6b presents the relative content of vimentin; the expression of vimentin in the anagen was higher than that in other periods, and the lowest expression was presented during telogen.

### Immunohistochemistry

**Fig. 7:**
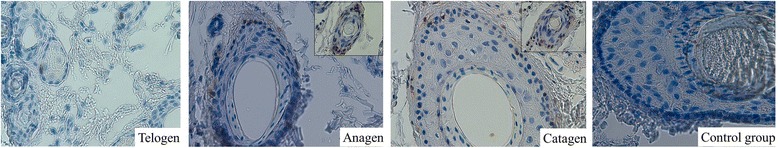
Localization of vimentin in the hair follicle cycle by immunohistochemistry. Brown cells were considered to be positive. The big picture is the primary hair follicle. The small picture is the secondary hair follicle in the corresponding month

Immunohistochemistry determined the expression sites of vimentin in the skin of Cashmere goat. These sites were observed under a light microscope, and brown cells were considered to be positive. The immunohistochemistry staining showed that the background staining was colorless or light blue (Fig. 7), and negative results showed no yellow or brown staining. Conversely, the experimental group detected a distinct yellow or brown staining, indicating that the immunohistochemistry method exhibited a specific immune response to vimentin (Fig. 7). The primary expression of vimentin was observed in the outer root sheath (ORS) of the hair follicle. As can be seen from Fig. 7, vimentin was expressed in the ORS in anagen and catagen, and no expression was found in telogen.

## Discussion

### The growth cycle of skin follicles in Inner Mongolia cashmere goats

The structure of the hair follicle is composed of a connective tissue sheath, ORS, inner root sheath (IRS), and a hair fiber from the outside to the inside [24]. After the birth of the animal, the skin follicles undergo periodic changes, and the periodic growth of the hair follicle is a complex and highly coordinated system of interaction between epithelial and dermal layers [25]. Previous studies [26, 27] showed that in anagen, the epithelial cells of the hair germ grow downwards, as an epidermal finger, into the dermis. After the predetermined depth is reached, the cells in the central cylinder reverse their growth direction and progress distally, forming the IRS and the hair shaft. Consecutively, the outer cells form the ORS and the dermal sheath. In catagen, the proliferation of the dermal papilla cells is decreased, the hair bulb atrophied, the hair papilla separated from other structures, and the hair follicle shortened. The telogen is not a vital process of cell proliferation, apoptosis, and differentiation. The paraffin section technique was used by Li et al. [28] to study the morphological changes of the Inner Mongolia Cashmere goat skin follicles. The study found that anagen occurred from April-November, catagen from December–January, and telogen from February-March, which indicates the duration of 8, 2, and 2 months, respectively. In this study, transcriptome sequencing and liquid chromatography-tandem mass spectrometry were used to obtain the nucleic acid and amino acid sequences of vimentin, and the comparison analysis used for the amino acid and nucleic acid sequences. RT-PCR, Western blot, and immunohistochemistry analyzed the expression level and expression sites of vimentin during the telogen, anagen, and catagen stages of the skin in Inner Mongolia Cashmere goat.

### Analysis of sequences and expression level of *VIM* gene and vimentin

Vimentin is a type III IF protein, which might be secreted by invading macrophages [29] or derived from the adjacent dermal telocytes [30]. The primary function of vimentin was to maintain the cell morphology [6–8], transmit signals between cells [9–11], and regulate the cell differentiation and proliferation [12]. In addition, vimentin induces cell migration [31]. The cell culture experiments revealed that the presence of vimentin in fibroblasts was associated with α-smooth muscle actin and myosin, and thus, the in vitro cultured fibroblasts also exhibit vimentin expression [32]. Notably, in the embryonic period, vimentin might participate in the adjustment of the arrangement of collagen fibers and inhibition of scar hyperplasia [33]. Furthermore, it is also involved in wound healing [34]. A previous study showed the highest expression of vimentin in the anagen of the growth cycle of the mouse skin follicle [21]. Another study revealed that vimentin was expressed at a maximum in the anagen of the Inner Mongolia Cashmere goat skin [22]. In the current study, the nucleic acid and amino acid sequences of vimentin in Inner Mongolian Cashmere goat skin sample were 100% identical to that of the *Capra hircus*. However, the rates of similarity to *Ovis aries*, *Bos taurus*, *Homo sapiens*, and *Mus musculus* were 99, 99, 98, and 97%, respectively. These results did not show any difference among the varieties of vimentin and only a little difference was observed among the species. Thus, vimentin is highly conserved, as postulated by Ivaska et al. [5]. Furthermore, we investigated the expression of the *VIM* gene and vimentin protein by RT-PCR and Western blot, respectively. The trend of expression of *VIM* and vimentin was increased significantly in anagen, which was in agreement with previous studies [21, 22]. A three-dimensional analysis of the structure might provide an insight into the relationship between the structure and function of vimentin in future studies.

### Localization analysis of vimentin in the hair follicles

Figure 4 demonstrated that vimentin was expressed only in the ORS of hair follicles. Therefore, the changes in the expression of vimentin and *VIM* genes in the skin samples should come from the ORS of the hair follicles. Thus, these results suggested that vimentin was involved in the regulation of the hair follicle growth cycle by affecting the ORS.

## Conclusions

The sequence and comparative analysis of *VIM* gene and vimentin protein revealed that vimentin was highly conserved across evolution. However, RT-PCR and Western blot demonstrated that the expression of *VIM* gene and vimentin increased with the growth of skin follicle and decreased with the decline in skin follicle growth. Immunohistochemistry showed that vimentin was expressed in the ORS of the hair follicle. Thus, we speculated that vimentin plays a role in the regulation of the hair follicle growth cycle by affecting the ORS.

## Abbreviations

BSA: Albumin from bovine serum
H2O2: Hydrogen peroxide
IF: Intermediate filament
IRS: Inner root sheath
ORS: Outer root sheath
PBS: Phosphate-buffered saline
PBST: Phosphate-buffered saline with tween20
RT-PCR: Real-time quantitative PCR
SDS: Sodium dodecyl sulfate
SDS-PAGE: SDS-polyacrylamide gel electrophoresis
